# Impact of an aerosolized or intramuscular adenovirus type 5-vectored COVID-19 vaccine on Fc-mediated immune effector functions in a hybrid immunity population

**DOI:** 10.3389/fimmu.2025.1657235

**Published:** 2025-10-01

**Authors:** Mingzhi Gan, Weiwei Han, Chuang Li, Simin Li, Zhuangzhuang Huang, Lingjie Xu, Xiaoyu Xu, Xiangjun Zhai, Yuxin Chen, Jingxin Li

**Affiliations:** ^1^ School of Public Health, National Vaccine Innovation Platform, Nanjing Medical University, Nanjing, China; ^2^ Jiangsu Provincial Medical Innovation Center, National Health Commission Key Laboratory of Enteric Pathogenic Microbiology, Jiangsu Provincial Center for Disease Control and Prevention (Jiangsu Provincial Academy of Preventive Medicine), Nanjing, China; ^3^ Department of Laboratory Medicine, Nanjing Drum Tower Hospital Clinical College of Nanjing University of Chinese Medicine, Nanjing, Jiangsu, China; ^4^ School of Public Health, Southeast University, Nanjing, China; ^5^ Vazyme Biotech Co., Ltd, Nanjing, China; ^6^ Major Project Executive Office, Jiangsu Provincial Center for Disease Control and Prevention, Nanjing, Jiangsu, China; ^7^ Department of Laboratory Medicine, Nanjing Drum Tower Hospital, Nanjing University Medical School, Nanjing, Jiangsu, China

**Keywords:** COVID-19, Fc-mediated immune effector functions, ADCP, ADNP, ADCC

## Abstract

**Background:**

Beyond the role of neutralizing antibodies in protecting against SARS-CoV-2, Fc-mediated antibodies functions may offer additional immune defense. This study aimed to evaluate the Fc-mediated immune responses elicited by aerosolized and intramuscular Ad5-nCoV vaccines in a Chinese population with hybrid immunity.

**Methods:**

Serum samples were collected from the immunogenicity sub-cohort within a multicenter, partially randomized platform trial comparing aerosolized and intramuscular adenovirus type 5-vectored COVID-19 vaccine (Ad5-nCoV) boosters in adults in China. Participants were enrolled approximately six months after an Omicron wave in late 2022, and randomized to receive a booster dose with aerosolized or intramuscular Ad5-nCoV. Fc-mediated immune responses to wild-type and XBB.1.16 variant spike proteins were assessed by measuring antibody-dependent cellular phagocytosis (ADCP), antibody-dependent neutrophil phagocytosis (ADNP), and antibody-dependent cellular cytotoxicity (ADCC) before vaccination, and at 14 days, 3 months, 6 months post-booster. Correlations between Fc-mediated responses (ADCP, ADNP, ADCC) and neutralizing antibodies, IgG, and IgA levels responses were also analyzed.

**Results:**

Intramuscular Ad5-nCoV vaccination significantly induced Fc-mediated effector functions against wild-type spike protein, with peak responses at 14 days post-booster, including ADCP score of 107.21 (95% CI: 84.43-129.99), ADNP score of 133.96 (95% CI: 112.81-155.11), and ADCC fold induction of 9.64 (95% CI: 8.57-10.70). These responses gradually waned over time. In contrast, aerosolized Ad5-nCoV did not significantly enhance ADCP or ADNP, but did elicit notable ADCC responses, peaking at 3 months post-vaccination (fold induction: 7.85,95% CI: 6.66-9.04). Fc-mediated responses to XBB.1.16 were lower than those to the wild-type spike. Notably, the fold reductions in ADCP, ADNP, and ADCC against XBB.1.16 were less pronounced than the corresponding reduction in neutralizing antibody titers.

**Conclusions:**

Intramuscular Ad5-nCoV vaccination elicited robust ADCP, ADNP, and ADCC responses, while the aerosolized formulation primarily induced ADCC activity. Fc-mediated effector functions exhibited greater cross-reactivity against emerging variants compared to neutralizing antibodies, but correlated only weakly with neutralizing antibody titers.

## Introduction

Widespread vaccination against severe acute respiratory syndrome coronavirus 2 (SARS-CoV-2) has significantly reduced global morbidity and mortality associated with COVID-19 (1). Most current vaccines were developed based on the ancestral SARS-CoV-2 strain. However, the emergence of SARS-CoV-2 variants with immune escape properties has challenged the effectiveness of both vaccine-induced or infection-acquired humoral immunity, as these variants can partially or fully evade neutralizing antibodies (2). Beyond neutralization, antibodies exert additional antiviral effects through interaction between their Fc region and Fc receptors (FcRs) on immune cells (3, 4). These Fc-mediated effector functions, including antibody-dependent cellular cytotoxicity (ADCC), antibody-dependent cellular phagocytosis (ADCP), and antibody-dependent neutrophil phagocytosis (ADNP), might also play crucial role in antiviral function independent of direct neutralization (5, 6). However, the immunological and clinical relevance of Fc-mediated responses remains underexplored, as most COVID-19 vaccine studies have focused primarily on neutralizing antibody titers.

Fc-dependent immune responses have been shown to play a crucial role in protection against a wide range of viral infections, including influenza, HIV, and Ebola (3). In the context of SARS-CoV-2, vaccines have been demonstrated to elicit Fc-mediated effector functions that contribute to viral clearance. For instance, three-dose regimen of CoronaVac significantly induced ADCP and ADNP responses against Omicron subvariants (7). Similarly, mRNA-1273 vaccine generated spike-specific antibodies with robust FcR-binding capacity and functional activity, even when neutralization was diminished by viral evolution (8). Furthermore, Fc-mediated antibody function, particularly those involving Fcγ receptor (FcγR) engagement, have been linked to improved clinical outcomes in COVID-19 (9).

In China, over 90% of the population has completed the primary COVID-19 vaccination series (10), and a large-scale national survey reported that 82.4% of individuals have been infected with SARS-CoV-2 between December 2022 and February 2023 (11). This had led to widespread hybrid immunity. Recent evidence suggests that hybrid immunity enhances FcR-binding antibody responses compared to vaccination alone, potentially promoting stronger activation of macrophages and natural killer (NK) cells, and augmenting ADCP, ADNP, and ADCC activity (12). Previously, we demonstrated that a booster dose of aerosolized or intramuscular adenovirus type 5-vectored COVID-19 vaccine (Ad5-nCoV) could enhance the humoral immunity against SARS-CoV-2 variants in individuals with a hybrid immunity (13). However, whether these booster strategies can also elicit robust Fc-mediated immune functions against emerging variants remains unclear.

In this study, we evaluate Fc-mediated immune responses elicited by aerosolized and intramuscular Ad5-nCoV booster vaccines in individuals with hybrid immunity. Specifically, we assessed their capacity to induce ADCP, ADNP and ADCC activity against SARS-CoV-2 wild-type strain and XBB.1.16 variants. These findings may provide new perspectives into optimizing booster vaccination strategies for population with hybrid immunity.

## Materials and methods

### Study cohort and serum sample collection

We did a multicenter, partially randomized platform clinical trial (ClinicalTrials.gov identifier: NCT05855408), to assess the efficacy of aerosolized (inhaled, IH) versus intramuscular (IM) administration of a booster dose of an Ad5-nCoV in adults aged ≥18 years with hybrid immunity, which has been reported previously (13).

Briefly, eligible participants were adults ≥18 years, with or without underlying medical conditions. Other key inclusion criteria included ≥4 months since SARS-CoV-2 infection or confirmation of no prior infection, and ≥6 months since the last COVID-19 vaccination. Participants provided written informed consent and were randomly assigned to receive either IH or IM Ad5-nCoV. Exclusion criteria included: suspected COVID-19 symptoms at enrollment, a positive SARS-CoV-2 antigen test, receipt of a second booster dose, history of severe adverse vaccine reactions or anaphylaxis, and pregnancy or lactation.

The first 60 individuals from each vaccination group were included in the immunogenetic subcohort. Blood samples were collected at baseline (pre-booster), and on day 14, month 3, and month 6 post-vaccination for serum and peripheral blood mononuclear cell (PBMC) isolation.

The study was approved by the Ethics Committee of the Jiangsu Provincial Center for Disease Control and Prevention and conducted in accordance with the Declaration of Helsinki and Good Clinical Practice (GCP) guidelines.

### Measurement of the antibody responses

#### Fc-mediated effector functional assays

##### ADCP and ADNP assay

ADNP and ADCP assays were carried out as previously study described (7). Spike proteins from SARS-CoV-2 Wild-type (WT) (Vazyme, Cat# CG202, Nanjing, China) and Omicron XBB.1.16 (Vazyme, Cat# CG282, Nanjing, China) variants were used. Proteins were biotinylated using Sulfo-NHS-LC biotin (Thermo Fisher Scientific, cat# A39257, MA, USA), with excess biotin removed via Zeba Spin Desalting Columns (Thermo Fisher Scientific, cat# A44300, MA, USA). Botinylated antigens were then coupled to 1 μm yellow-green fluorescent NeutrAvidin beads (Invitrogen, cat# F8776, MA, USA) in a 1:1 ratio and incubated at 37°C for 2 hours, followed by centrifugation (16,000 g, 15 min) and resuspension in Phosphate-Buffered Saline (PBS) with 0.5% Bovine Serum Albumin (BSA).

For the ADCP assay, THP-1 cells were used. Serum samples were diluted 1:25 in PBS containing 0.5% Tween-20 and 1% BSA, and incubated with antigen-coupled beads at 4°C overnight. After washing, 25,000 THP-1 cells were added per well in R10 medium and incubated at 37°C, 5% CO_2_ for 1 hour. Cells were then washed with PBS and fixed with 4% paraformaldehyde (PFA) (Leagene, cat# DF0135, Beijing, China) for 30 min. The fixed cells were then analyzed by flow cytometry. Serum samples from healthy archived individuals from 2019, who were unexposed and unvaccinated, served as negative controls, which were assayed in parallel with the test samples.

For the ADNP assay, ADNP granulocytes were isolated from the whole blood of healthy adult donors using a lysis method (8). Immune complexes were prepared by incubating the 25-fold diluted serum with antigen-coupled beads (Invitrogen, cat# F8776, MA, USA), as described above. After incubation, unbound immunoglobulins were removed by washing with sterile PBS. Granulocytes were resuspended in R10 medium at a concentration of 5 00,000 cells/mL. A total of 50,000 cells were added to each well containing immune complexes, mixed by gentle shaking, and incubated for 1 hour at 37°C with 5% CO_2_. Following incubation, cells were washed and stained for neutrophil identification using an APC-conjugated anti-CD66b antibody (BioLegend, cat# 17-0666-42, CA, USA). Fixation was carried out using 4% PFA (Leagene, cat# DF0135, Beijing, China) for 30 minutes. Flow cytometric analysis was subsequently performed. The same negative control sera used in the ADCP assay were included and assayed in parallel with the test samples.

To assess the phagocytic efficacy, the geometric mean fluorescence intensity (gMFI) and percentage of bead-positive cells (THP-1 or neutrophils) were measured by flow cytometry. The phagoscore was calculated as: Phagoscore = (% positive cells × gMFI)/100,000. The corrected phagoscore was defined as the actual phagoscore derived from the sample minus phagoscore derived from the blank control. If the phagoscore was calculated to be less than zero, it was assigned a value of zero.

##### ADCC assay

ADCC assay was carried out as previously study described (14). Two sets of 293F cell lines were constructed, each stably expressing the spike protein of either the SARS-CoV-2 wild-type (WT) strain or the XBB.1.16 variant. These cell lines were established by transfecting the respective plasmids and selecting stable clones, which were then used as target cells in the ADCC assay. Jurkat-FcγRIII-NFAT-Luc reporter cells (Vazyme, cat# DD1301-1, Nanjing, China) were used as effector cells. These cells stably express the FcγRIIIa (CD16) receptor and contain a functional NFAT transcription factor that regulates the expression of the Lucia luciferase gene. When antibodies bind to FcγRIIIa, the NFAT pathway is activated, resulting in NFAT translocation to the nucleus and subsequent binding to the promoter of the Lucia luciferase gene, driving luciferase expression (15).

Serum samples were diluted 1:60 in R10 medium and incubated with 25,000 target 293F cells per well at 37°C and 5% CO_2_ for 1 hour. After incubation, 75,000 Jurkat reporter cells were added to each well, gently mixed, and further incubated under the same conditions for 12 hours. Luciferase substrate (Vazyme, cat# DD1201-03, Nanjing, China) was then added, and the wells were shaken to ensure a complete reaction. Relative light units (RLU) were measured using the protocol provided by PerkinElmer (Waltham, MA). Serum samples from healthy adult donors collected in 2019, identical to those used in the ADCP assay, were included as negative controls. ADCC activity was calculated as the fold induction of luciferase activity relative to the negative control sera.

#### Pseudovirus neutralization assay and IgG/IgA testing

Neutralizing antibody responses against wild-type SARS-CoV-2, XBB.1.16, and BA.4/5 variants were assessed using pseudovirus neutralization tests, employing a human immunodeficiency virus pseudovirus system expressing the spike glycoprotein. In the experiment, serum samples were incubated with pseudoviruses expressing the spike glycoprotein of the aforementioned variants, and then the mixture was added to HEK293-ACE2 cells. The viral infectivity was assessed by measuring the expression level of the reporter gene using the Bio-Lite luciferase reporter assay kit. The neutralizing antibody activity was quantified by calculating the half-maximal inhibitory concentration (IC50). To ensure sensitivity, the cutoff titer for detection was set at 1:30.

The RBD-specific IgG and IgA antibody responses were evaluated using ELISA kits from Vazyme Biotech (Nanjing, China). For IgG detection, a quantitative ELISA kit was used, where the optical density (OD) was calculated by subtracting the OD value at 630 nm from that at 450 nm. The lower limit of quantification for the IgG assay was 0.125 BAU/mL. The final concentration of IgG antibodies was determined by multiplying the detected OD value by the serum dilution factor. IgG antibodies were assessed against both wild-type (WT) SARS-CoV-2 and BA.4/5 variants. For IgA detection, an indirect ELISA method was used with the RBD-specific IgA ELISA kit. After a two-step incubation and color development, the absorbance at a specific wavelength was positively correlated with the concentration of SARS-CoV-2 RBD IgA antibodies targeting the XBB.1.5 variant. The antibody concentration was then calculated by referencing a standard curve.

### Statistical analysis

The χ²-test or Fisher’s exact test was used for categorical demographic data. Student’s t-test was used for continuous demographic data. For comparisons of ADCP, ADNP, and ADCC responses at different time points, mixed-effect models was performed, followed by Tukey’s correction for multiple comparisons. Single-variable comparisons between the two vaccine groups, were conducted using the Mann-Whitney U test. Spearman correlation analysis was used to evaluate the relationships between ADCP, ADNP, ADCC, NAb, IgG, and IgA. A correlation heatmap was generated using ChiPlot (https://www.chiplot.online/correlation_heatmap.html). For comparisons of ADCP, ADNP, ADCC, NAbs, and IgG responses specific to the COVID-19 wild-type strain versus Omicron subvariants, paired t-tests were performed. Data analysis was carried out using SPSS version 26 and GraphPad Prism version 9.5.1. All statistical tests were two-tailed, and a p-value of < 0.05 was considered statistically significant.

## Results

### Longitudinal Fc-mediated effector functions to WT and XBB.1.16 spikes in two vaccination groups

We assessed Fc-mediated effector functions (ADCP, ADNP, and ADCC) in 121 participants (60 from the IM Ad5-nCoV group and 61 from the IH Ad5-nCoV group) at four time points: baseline (pre-booster), day 14 (14 days post-booster), month 3 (3 months post-booster), and month 6 (6 months post-booster). There were no significant differences between groups in demographic characteristics, including age, sex, BMI and vaccination history (**Table 1**).

**Table 1:** Demographic characteristics of the participants in this study.

Characteristic	IM Ad5-nCoV (n=60)	IH Ad5-nCoV (n=61)	P value
Age, years			
Mean (SD)	47.33(13.4)	47.85(13.8)	0.834
Sex, n (%)			
Female	40(66.7)	36(59.0)	0.384
Male	20(33.3)	25(41.0)	
BMI, kg/m^2^			
Mean (SD)	24.3(3.8)	24.1(3.1)	0.660
Vaccination history before booster, n (%)			
ICV+ICV+ICV	58(96.7)	60(98.4)	0.619
ICV+ICV	1(1.7)	0(0.0)	
ICV+ICV+Ad5-IM	0(0.0)	1(1.6)	
CHO+CHO+CHO	1(1.7)	0(0.0)	

Data are n (%) or mean (SD). BMI=Body-mass index. IM Ad5-nCoV (Ad5-IM) =adenovirus type 5 vectored COVID-19 vaccine through intramuscular injection. IH Ad5-nCoV=adenovirus type 5 vectored COVID-19 vaccine through oral inhalation. ICV=inactivated COVID-19 vaccine. CHO=SARS-CoV-2 recombinant protein vaccine (CHO cell).

**Figure 1:**
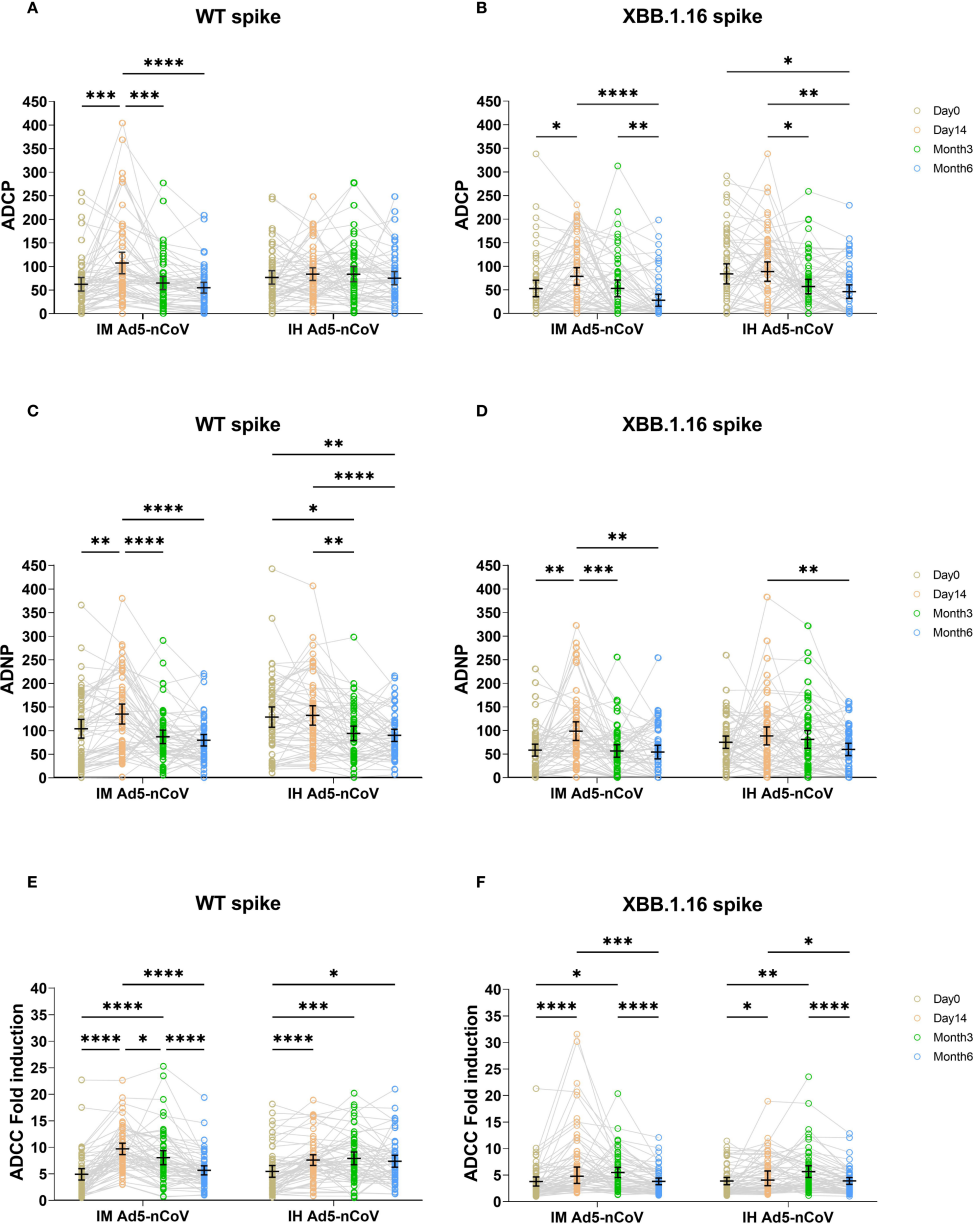
ADCP, ADNP, and ADCC specific to WT and XBB.1.16 spike proteins across four time points in two vaccination groups. ADCP **(A, B)**, ADNP **(C, D)**, and ADCC **(E, F)** specific to WT and XBB.1.16 spike proteins were measured before the booster dose, and at day 14, month 3, and month 6 post-booster. Trends over time were compared across the two groups using two-sided mixed-effects models, followed by Tukey’s correction for multiple comparisons. IM Ad5-nCoV=adenovirus type 5 vectored COVID-19 vaccine through intramuscular injection. IH Ad5-nCoV=adenovirus type 5 vectored COVID-19 vaccine through oral inhalation. Significant differences are indicated by asterisks: *p < 0.05, **p < 0.01, ***p < 0.001, ****p <0.0001.

In the IM Ad5-nCoV group, WT spike-specific ADCP significantly increased at day 14, with a mean phagoscore of 107.21 (95% CI: 84.43-129.99) compared to baseline (62.15, 95% CI: 47.82-76.48). However, ADCP responses declined by month 3 (64.65, 95% CI: 50.78-78.51) and further by month 6 (54.77, 95% CI: 43.38-66.15). In contrast, IH Ad5-nCoV induce no significant changes in ADCP responses over time, with phagoscores remaining stable at 76.72 (95% CI: 62.47-90.96) at baseline, 83.70 (95% CI: 70.19-97.20) at day 14, 83.55 (95% CI: 67.41-99.70) at month 3, and 75.12 (95% CI: 65.21-89.04) at month 6 (**Figure 1A**). In contrast to ADCP responses against wild-type SARS-CoV-2, those against XBB.1.16 were observed at lower levels across all time points, with the peak phagoscore at day 14 of 78.84 (95% CI: 60.35-97.32) in IM Ad5-nCoV group. Similarly, IH Ad5-nCoV did not induce significant changes in ADCP responses against XBB.1.16, with responses showing a gradual decline at month 3 and month 6 (**Figure 1B**).

IM Ad5-nCoV significantly induced ADNP responses for WT Spike, with phagoscores of 102.81 (95% CI: 82.97-122.65) at baseline, 133.96 (95% CI: 112.81-155.11) at day 14, and followed by a gradual decline at month 3 (85.64, 95% CI: 71.30-99.98) and month 6 (78.42, 95% CI: 66.29-90.55). In comparison, IH Ad5-nCoV did not elicit a significant enhancement in ADNP responses. A significant decline was observed over time, with the mean phagocytic score decreasing to 92.90 (95% CI: 77.62-108.17) at month 3 and 88.86 (95% CI: 75.96-101.77) at month 6, relative to the response level on day 14 post-vaccination (131.0, 95% CI: 110.4-151.7). (**Figure 1C**). For the XBB.1.16 Spike, a similar trend was observed. In contrast to ADNP responses against wild-type SARS-CoV-2, those against XBB.1.16 were observed at lower levels across all time points, with the peak phagoscore at day 14 of 97.61(95% CI: 77.80-117.41) in IM Ad5-nCoV group. Similarly, IH Ad5-nCoV did not elicit significant ADNP responses against XBB.1.16, and a significant decline was observed at month 6 (58.94,95% CI: 45.80-72.07) compared to day 14 (87.47, 95% CI: 68.47-106.47). (**Figure 1D**).

Both vaccines induced ADCC responses against WT spike. IM Ad5-nCoV vaccination induced a peak response at day 14 (9.64-fold, 95% CI: 8.57-10.70), which declined by month 3 (7.98, 95% CI: 6.66-9.30) and returned to near-baseline levels by month 6 (5.60, 95% CI: 4.73-6.46). By comparison, IH Ad5-nCoV elicited more durable ADCC responses, peaking at month 3 (7.85, 95% CI: 6.66-9.04) and remaining stable at month 6 (7.30, 95% CI: 6.17-8.42) (**Figure 1E**). In contrast to ADCC responses against wild-type spike, ADCC responses against XBB.1.16 followed a similar kinetics pattern, albeit with lower magnitudes (**Figure 1F**). Specifically, IM Ad5-nCoV group generated a peak fold increase at day 14 (7.22,95% CI: 5.51-8.92), whereas the IH Ad5-nCoV group peaked at month 3 (5.67, 95% CI: 4.57-6.77) followed by a significant decline at month 6 (3.90, 95% CI: 3.26-4.54).

### IM Ad5-nCoV induces more pronounced Fc-mediated effector functions than IH Ad5-nCoV

To account for baseline variability, the Fc-mediated immune effector functions at day 14, month 3, and month 6 were standardized by calculating fold changes relative to each individual’s own baseline, with pre-booster levels used for comparison. After standardization, we compared the ADCP, ADNP, and ADCC responses across these three time points. For ADCP and ADNP responses against both WT and XBB.1.16 strains, IM Ad5-nCoV group and IH Ad5-nCoV group showed a decrease at month 3 relative to day 14, with further decline at month 6 (**Supplementary Figures S1A, B**). In the case of ADCC responses to both WT and XBB.1.16 strains, IM Ad5-nCoV group exhibited a decrease at month 3 relative to day 14, with further reduction at month 6. However, for the WT spike-specific ADCC response, IH Ad5-nCoV group did not show a downward trend, whereas for XBB.1.16 variant, the response at month 6 was lower than a month 3 (**Supplementary Figure S1C**). These findings confirm that the trends observed in both raw and standardized data are consistent.

**Figure 2:**
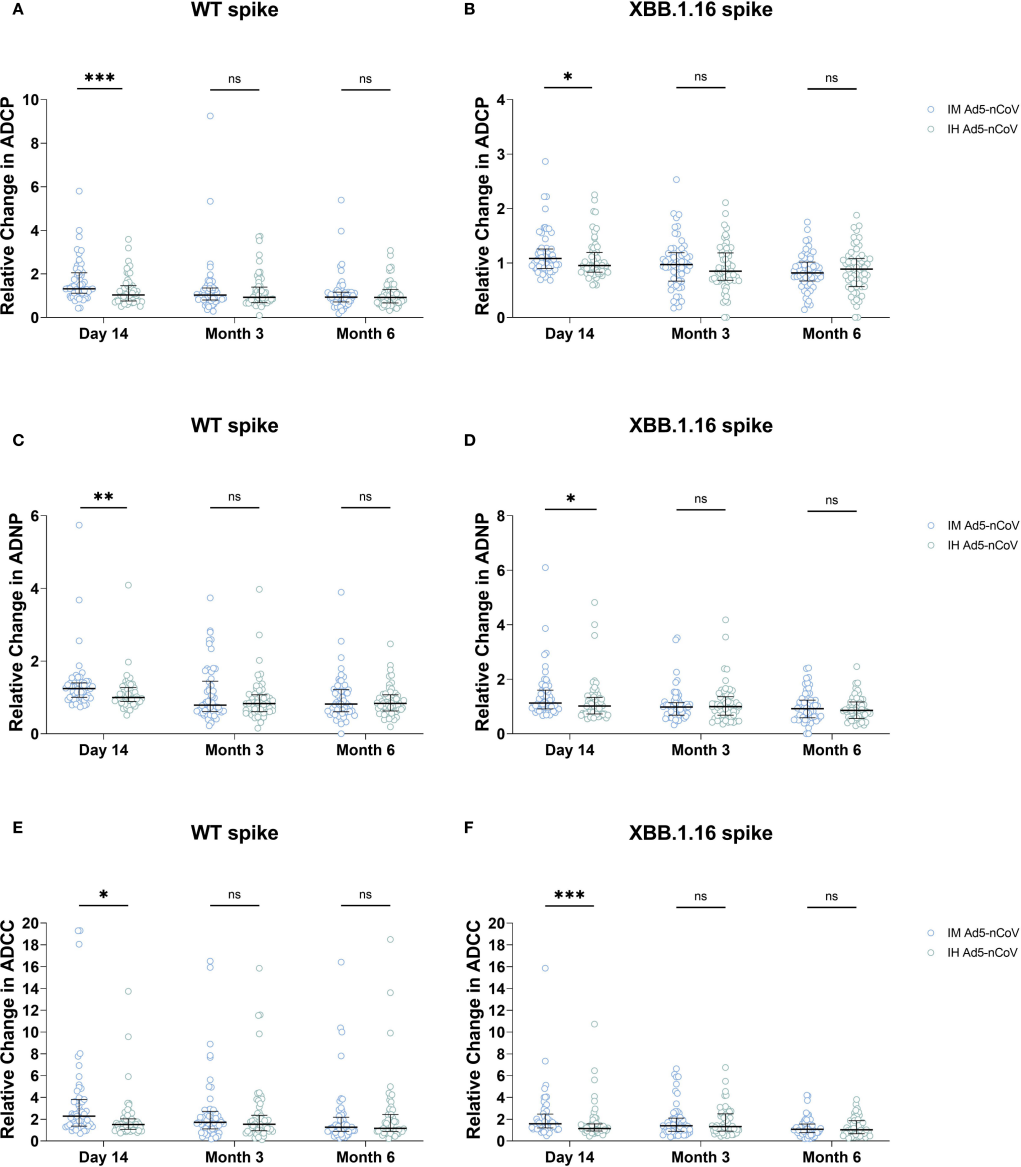
Fold-change comparison of ADCP, ADNP, and ADCC responses between two vaccines at three time points. Panels show the relative change in **(A, B)** ADCP, **(C, D)** ADNP, and **(E, F)** ADCC responses specific to the SARS-CoV-2 wild-type (WT) spike **(A, C, E)** and XBB.1.16 spike **(B, D, F)** at day 14, month 3, and month 6 post-booster, compared between the IM Ad5-nCoV and IH Ad5-nCoV groups. Statistical comparisons between the two vaccine groups at each time point were performed using the Mann-Whitney U test. IM Ad5-nCoV=adenovirus type 5 vectored COVID-19 vaccine through intramuscular injection. IH Ad5-nCoV=adenovirus type 5 vectored COVID-19 vaccine through oral inhalation. Asterisks indicate statistically significant differences between the groups at each time point: *p < 0.05, **p < 0.01, ***p < 0.001. ns indicates no significant difference.

Then, we compared the fold changes in Fc-mediated immune effector functions between the two vaccines. Against WT spike, IM Ad5-nCoV induced 1.85-fold greater ADCP responses at day 14, compared to 1.22-fold increase for IH Ad5-nCoV (*P* = 0.0003). No significant differences were observed between the two vaccines at month 3 and month 6(**Figure 2A**). For the XBB.1.16 spike, a modest but significant improvement was observed at day 14 (1.17 vs. 1.06, *P* = 0.047) (**Figure 2B**), with no significant differences at later timepoints.

A similar pattern was observed for ADNP responses. IM Ad5-nCoV also induced 1.34-fold increase for wild-type spike specific ADNP responses at day 14 versus 1.11 for IH Ad5-nCoV group (*P* = 0.002), and there was no significant differences were observed at month 3 and month 6 (**Figure 2C**). For the XBB.1.16 spike, the fold increase for ADNP responses at 14 days was 1.41 for IM Ad5-nCoV versus 1.20 for IH Ad5-nCoV (*P* = 0.023), with no significant differences at month 3 and month 6 (**Figure 2D**).

Similarly, IM Ad5-nCoV also outperformed IH Ad5-nCoV in ADCC responses. For the WT spike, the mean fold increase at day 14 was 3.86 for IM Ad5-nCoV versus 2.03 for IH Ad5-nCoV (*P* = 0.001), with no significant differences at later timepoints (**Figure 2E**). For the XBB.1.16 spike, the mean fold increase in ADCC at day 14 was 2.20 for IM Ad5-nCoV versus 1.65 for IH Ad5-nCoV (P = 0.001). There were no statistical differences at subsequent timepoints (**Figure 2F**).

### Correlation between Fc-mediated immune effector functions and antibody responses

We previously analyzed the levels of IgG, IgA, and neutralizing antibodies (NAbs) in both cohorts (13). IH Ad5-nCoV group showed peak NAbs against wild-type SARS-CoV-2 at month 3 (GMT:1026.2, 95% CI 792.7-1328.6) and slightly declined at month 6 (880.9, 95% CI 700.9-1107.2). In comparison, the IM Ad5-nCoV group exhibited similar levels at day 14 (796.4, 95% CI 635.3-998.2), but decreased at month 3 (681.6, 95% CI 542.2-856.9) and month 6 (520.0, 95% CI 413.1-654.6) (**Supplementary Figure S2A**). Both groups showed comparable NAbs against BA.4/5 (**Supplementary Figure S2B**), with the peak GMTs at month 3 of 1061.0 (95% CI 800.1-1405.5) in the IH Ad5-nCoV group and 883.0 (95% CI 670.1-1163.4) in the IM Ad5-nCoV group. However, NAbs against XBB.1.16 were lower in both groups compared to WT SARS-CoV-2 and BA.4/5 variants, though GMTs significantly increased post-booster (**Supplementary Figure S2C**).

RBD-specific IgG responses followed a similar pattern to NAbs. WT-specific IgG geometric mean concentrations (GMCs) rose from 1071.2 (95% CI: 833.1-1377.3) to 2174.1 (95% CI: 1890.4-2500.5) BAU/mL at day 14 in the IM Ad5-nCoV group, and from 1635.9 (95% CI: 1339.0-1998.7) to 2779.8 (95% CI: 2432.1-3177.2) BAU/mL at month 3 in the IH Ad5-nCoV group (**Supplementary Figure S3A**). Both vaccines significantly increased IgG against BA.4/5 despite of modest magnitude (**Supplementary Figure S3B**). Additionally, IgA antibodies against the XBB.1.5 variant peaked at month 3 post-vaccination in IM Ad5-nCoV group, followed by a slight decline at month 6, with the peak GMCs at month 3 of 456.9 (95% CI: 296.9-615.8) U/mL, and the IH Ad5-nCoV group showed a peak at day 14 of 477.4 (364.7-625.0) U/mL, which decreased by month 3 and declined further by month 6 (**Supplementary Figure S4**).

**Figure 3:**
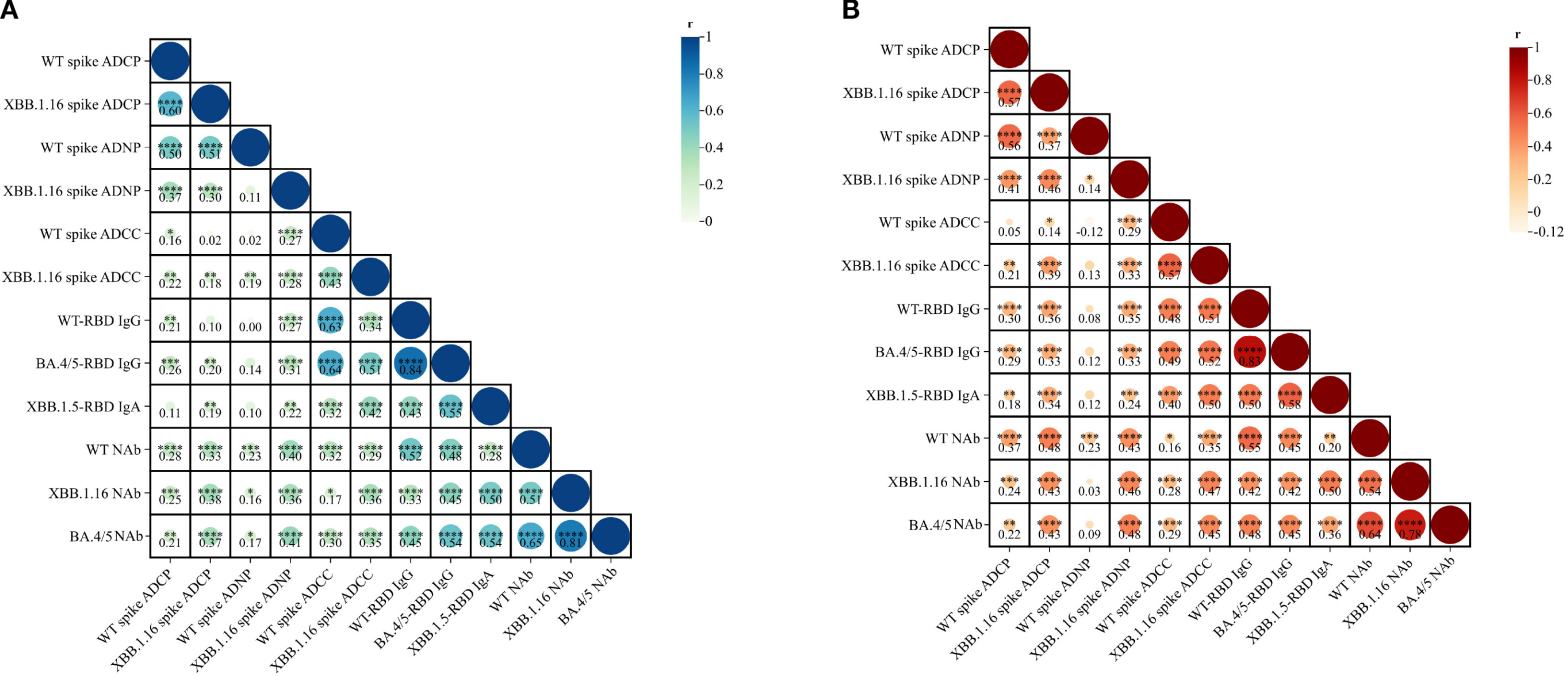
Correlation analysis of ADCP, ADNP, ADCC, IgG, IgA, and neutralization activity elicited by IM Ad5-nCoV **(A)** and IH Ad5-nCoV **(B)**. Statistics were analyzed using Spearman correlation analysis. Spearman correlation coefficients (r) are displayed within the matrix, with significant correlations indicated by asterisks (*p < 0.05, **p < 0.01, ***p < 0.001, ****p < 0.0001). IM Ad5-nCoV=adenovirus type 5 vectored COVID-19 vaccine through intramuscular injection. IH Ad5-nCoV=adenovirus type 5 vectored COVID-19 vaccine through oral inhalation.

Spearman correlation analysis showed moderate correlations between ADCP and ADNP for the wild-type spike (IM: *r* = 0.50; IH: r = 0.56, both *P* < 0.0001) and XBB.1.16 spike (IM: *r* = 0.30; IH: *r* = 0.46, both *P* < 0.0001). Nevertheless, ADCC correlated weakly with ADCP and ADNP. Correlation between Fc effector functions and antibody levels were generally weak for ADCP/ADNP, and IgG, but moderate-to-strong for ADCC and IgG. Specifically, WT spike-specific ADCC was correlated with WT RBD-specific IgG antibodies (*r* = 0.63 in IM, r=0.48 in IH, both *P* < 0.0001), and also correlated with BA.4/5 RBD-specific IgG (IM: r = 0.64; IH: r = 0.49; both *P* < 0.0001). For the XBB.1.16 spike, ADCC in the IH group demonstrated a moderate correlation with WT RBD-specific IgG antibodies (r = 0.51; *P* < 0.0001), whereas the correlation in the IM group was weaker (r = 0.34; *P* < 0.0001). Correlations between ADCC and BA.4/5 RBD-specific IgG antibodies were moderate in both groups (IM: r = 0.51; IH: r = 0.52; both *P* < 0.0001). IgA correlated moderately with ADCC responses, but weakly with ADCP and ADNP. In particular, XBB 1.16 spike-specific ADCC effects exhibited a moderate positive correlation with XBB.1.5 RBD-specific IgA antibodies (*r* = 0.42 in IM, *r* = 0.50 in IH, both *P* < 0.0001). Correlation with NAbs were weak across all Fc functions in both vaccine groups (**Figures 3A, B**).

### Fc-mediated effector functions demonstrate stronger cross-reactivity than the IgG and neutralizing antibodies

**Figure 4:**
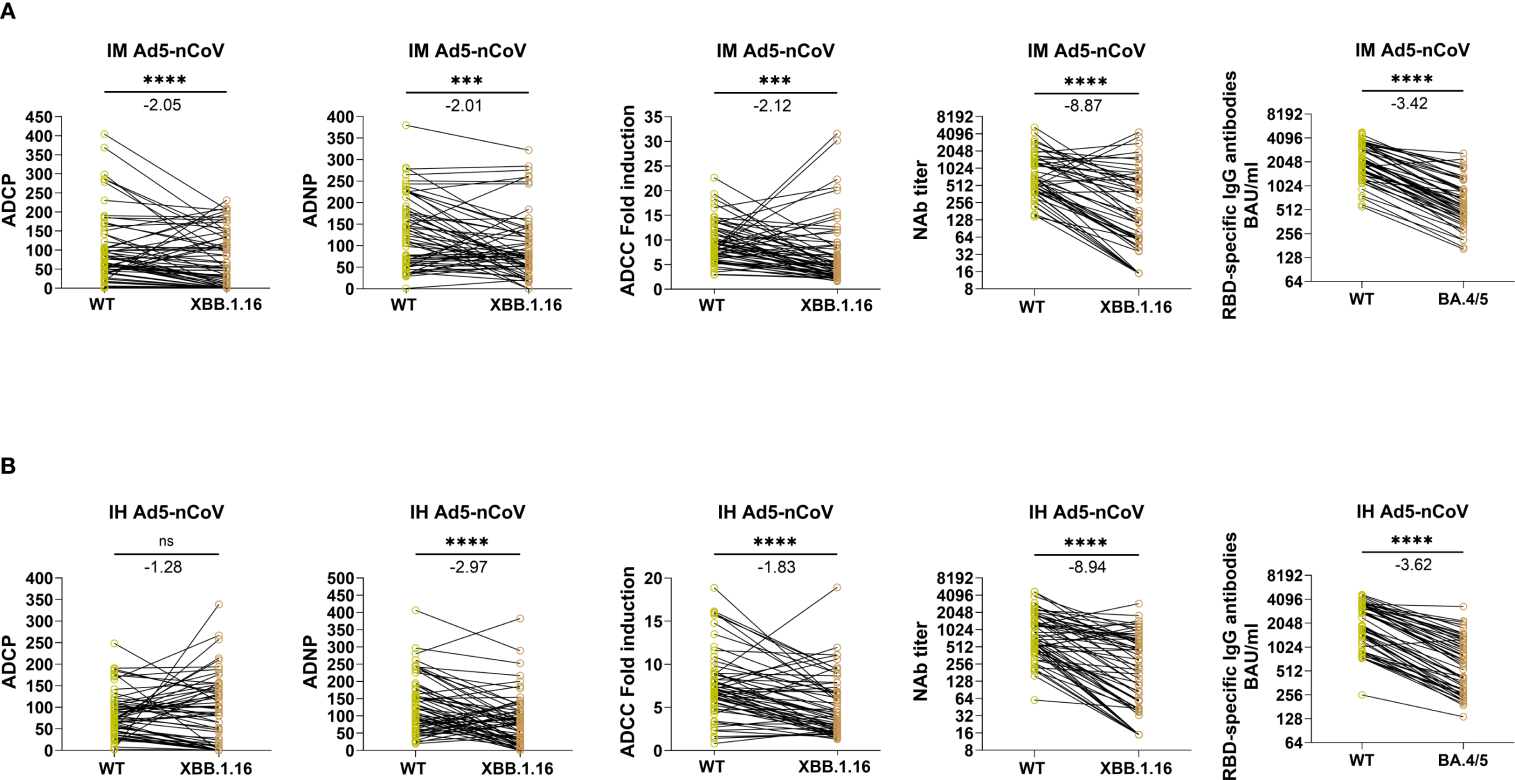
Paired comparisons of Fc-mediated effector functions and antibody responses to SARS-CoV-2 wild-type and Omicron subvariants at day 14 following IM Ad5-nCoV or IH Ad5-nCoV vaccination. Panels **(A, B)** present data from IM and IH Ad5-nCoV groups, respectively. Immune responses to the WT and Omicron variant spikes were compared at day 14 post-booster, including ADCP, ADNP, ADCC, NAb titers, and RBD-specific IgG. Statistical analyses were performed using the Wilcoxon matched-pairs signed-rank test. Fold reductions in immune responses from WT to variant were calculated for each individual, and the group mean values are annotated above each plot. IM Ad5-nCoV=adenovirus type 5 vectored COVID-19 vaccine through intramuscular injection. IH Ad5-nCoV=adenovirus type 5 vectored COVID-19 vaccine through oral inhalation. Asterisks indicate statistical significance: ***p < 0.001, ****p < 0.0001. ns indicates no significant difference.

We further compared the fold reduction in Fc effector function and NAbs from WT spike to XBB.1.16 spike as well as spike-specific IgG responses from WT to BA.4/5. In the IM Ad5-nCoV group (**Figure 4A**), the GMT of WT-specific NAb dropped from 796.4 (95% CI: 635.3-998.2) to 201.3 (95% CI: 130.5-310.6) against XBB.1.16 (8.87-fold reduction, *P* < 0.0001). The GMC of WT-specific IgG decreased from 2174.1 (95% CI: 1890.4-2500.5) BAU/mL to 632.4 (95% CI: 527.1-758.8) BAU/mL (3.42-fold, *P* < 0.0001). In contrast, the fold reduction was remarkably smaller for ADCP, ADNP and ADCC. The mean phagocytic score of WT-specific ADCP dropped from 107.21 (95% CI: 84.43-129.99) to 78.84 (95% CI: 60.35-97.32) against XBB.1.16 (2.05-fold, *P* < 0.0001). The mean phagocytic score of WT-specific ADNP dropped from 133.96 (95% CI: 112.81-155.11) to 97.61 (95% CI: 77.80-117.41) against XBB.1.16 (2.01-fold, *P* = 0.0008). The mean fold induction of WT-specific ADCC dropped from 9.64 (95% CI: 8.57-10.70) to 7.22 (95% CI: 5.51-8.92) against XBB.1.16 (2.12-fold, *P* = 0.0005).

Similarly, in the IH Ad5-nCoV group (**Figure 4B**), the GMT of WT-specific NAb dropped from 796.9 (95% CI: 635.6-999.1) to 170.5 (95% CI: 114.7-253.4) against XBB.1.16 (8.94-fold reduction, *P* < 0.0001). The GMC of WT-specific IgG decreased from 2779.8 (95% CI: 2432.1-3177.2) BAU/mL to 589.3 (95% CI: 478.5-725.8) BAU/mL against BA.4/5 (3.62-fold, *P* < 0.0001). Again, the fold reduction in Fc-mediated functions was considerably smaller. The mean phagocytic score for WT-specific ADCP remained almost unchanged, from 83.70 (95% CI: 70.19-97.20) to 88.94 (95% CI: 68.5-109.4) against XBB.1.16 (1.28-fold, *P* = 0.54). The mean phagocytic score of ADNP dropped from 131.0 (95% CI: 110.4-151.7) to 87.5 (95% CI: 68.5-106.5), a 2.97-fold reduction (*P* < 0.0001). The mean fold induction of ADCC dropped from 7.5 (95% CI: 6.5-8.5) to 5.1 (95% CI: 4.2-6.0), a 1.83-fold reduction (*P* < 0.0001). Collectively, these findings indicate that Fc-mediated effector functions retain broader cross-reactivity against emerging variants compared to NAb and IgG responses.

## Discussion

In this study, we demonstrate that intramuscular administration of Ad5-nCoV booster, based on the ancestral strain, elicits robust Fc-mediated effector functions, with ADCP, ADNP, and ADCC responses peaking at 14 days post-boost. These findings align with previous studies reporting that both inactivated and mRNA vaccines encoding the ancestral spike protein can effectively induce potent Fc-dependent immune responses (12, 14).

While the aerosolized Ad5-nCoV vaccine effectively induced ADCC responses, it showed limited capacity to stimulate ADCP and ADNP activities. Furthermore, Fc-mediated effector functions were consistently lower in IH group than IM group. This discrepancy may be attributable to the distinct immunological microenvironments and mechanisms active by two delivery routes. Aerosolized vaccines primarily stimulate mucosal immunity in the respiratory tract, whereas intramuscular vaccination induces more robust systemic immunity (16). Of note, systemic vaccination triggers strong cytokine responses, including IFN-γ, TNF-α, and IL-2 from activated immune cells. Of particular importance is IFN-γ, which enhances macrophage and NK cell function and upregulates Fc receptor expression, thereby amplifying Fc-mediated immune activity (17). These immunological mechanisms likely underpin the superior Fc-effector responses observed following IM vaccination. In addition, our results showed that in the IH group, XBB.1.16 spike-specific ADCP and WT spike-specific ADNP at month 6 post-booster declined to below baseline levels. A plausible explanation is that at baseline, participants were in a state of hybrid immunity. Previous studies have shown that hybrid immunity elicits stronger Fc-mediated effector functions compared with vaccination alone (12, 18). By contrast, the aerosolized Ad5-nCoV booster failed to induce robust ADCP and ADNP responses, which likely contributed to the sub-baseline levels observed at 6 months post-booster in the IH group.

We also observed only weak to moderate correlations between Fc-mediated effector functions and levels of NAbs, IgG or IgA. This finding is consistent with prior studies, including those identifying ADCC responses in convalescent individuals post SARS-CoV-2 infection (18). The spike glycoprotein remains the primary target of SARS-CoV-2 antibody responses, with dominant neutralizing epitopes localized in the RBD of the S1 subunit (19). However, evidence suggests that, in hybrid immunity, antibodies targeting the S2 subunit can exhibit enhanced Fc receptor-binding capacity and more effectively mediate immune cell activation, particularly via ADCP, ADNP, and ADCC responses (12). This functional divergence may explain the limited correlation between Fc effector functions and conventional humoral markers. Indeed, Fc activity appears to reply more on the structural properties of the antibody Fc domain than on neutralizing potency, suggesting that even antibodies with limited neutralizing capacity may provide meaningful protection via Fc-driven mechanisms.

Our data also provide important insights into cross-variant immune responses. Previous work has shown that infection with Beta and Delta variant induces more broadly cross-reactive Fc-mediated antibodies, compared to infection with D614G strain or Ad26.COV2.S vaccination (20). Similarly, in our study, ADCP, ADNP, and ADCC responses against XBB.1.16 variant, while lower than those against WT spike, declined less sharply than NAbs and IgG levels. These observations echo the finding from mRNA-1273 vaccine studies, which demonstrated the sustained Fc-mediated immunity across a wide range of SARS-CoV-2 variants (8). The broader Fc cross-reactivity might be explained by two principal factors. First, most mutations in variants of concerns (VOCs) cluster in the RBD and N-terminal domain (NTD), limiting their impact on Fc-epitope recognition (21). Secondly, Fc-mediated functions are less affected by mutations that alter ACE2 receptor binding, which primarily influence neutralization capacity (22, 23).

This study has several limitations. First, we focused on Fc-mediated effector responses against the XBB.1.16 variant, and did not assess other currently circulating variants. Second, all vaccines administered targeted the ancestral strain, precluding direct comparison with variant-adapted vaccines. Third, the follow up duration was limited to six months post-vaccination, restricting conclusions regarding the long-term immune durability. Finally, the relatively small cohort size (n=121) may limit the generalizability across broader populations.

In conclusion, this study provides a comprehensive evaluation of Fc-mediated immune responses following IM and IH administration of Ad5-nCoV. Our findings show that IM vaccination induces significantly stronger ADCP, ADNP, and ADCC activity, while IH vaccination effectively elicits ADCC responses. Importantly, Fc-mediated effector functions displayed broader cross-reactivity against emerging variants compared to NAbs and IgG responses. These results not only underscore the pivotal role of Fc effector functions in vaccine-mediated immunity, but also highlight the necessity of optimizing Fc-mediated immunity in future vaccine design strategies, particularly against rapidly evolving viral pathogens.

## Data Availability

Individual participant data underlying the results reported in this article are available under restricted access due to requirements imposed by the Chinese Human Genetic Resources Administration regarding the public disclosure of clinical trial data. Researchers who provide a scientifically sound proposal will be allowed access to the de-identified individual participant data. Individual participant data can be obtained by submitting a request to the corresponding authors.
