# Supplementary material for: Impact of an aerosolized or intramuscular adenovirus type 5-vectored COVID-19 vaccine on Fc-mediated immune effector functions in a hybrid immunity population
Source: Front Immunol. 2025 Oct 1;16:1657235. doi: 10.3389/fimmu.2025.1657235 (PMC12521264; doi:10.3389/fimmu.2025.1657235)

**Supplementary Figures**

Figure S1. **Fold-change comparison of ADCP, ADNP, and ADCC specific to WT and XBB.1.16 spike proteins across three time points in two vaccination groups.** The relative change in ADCP(A), ADNP(B), and ADCC(C) specific to WT and XBB.1.16 spike proteins were measured at day 14, month 3, and month 6 post-booster. Data are represented as fold change relative to pre-booster levels. Statistical significance was assessed using the Friedman test. The p values for multiple comparisons have been adjusted. IM Ad5-nCoV=adenovirus type 5 vectored COVID-19 vaccine through intramuscular injection. IH Ad5-nCoV=adenovirus type 5 vectored COVID-19 vaccine through oral inhalation. Significant differences are indicated by asterisks: * p < 0.05, ** p < 0.01, *** p < 0.001, **** p <0.0001


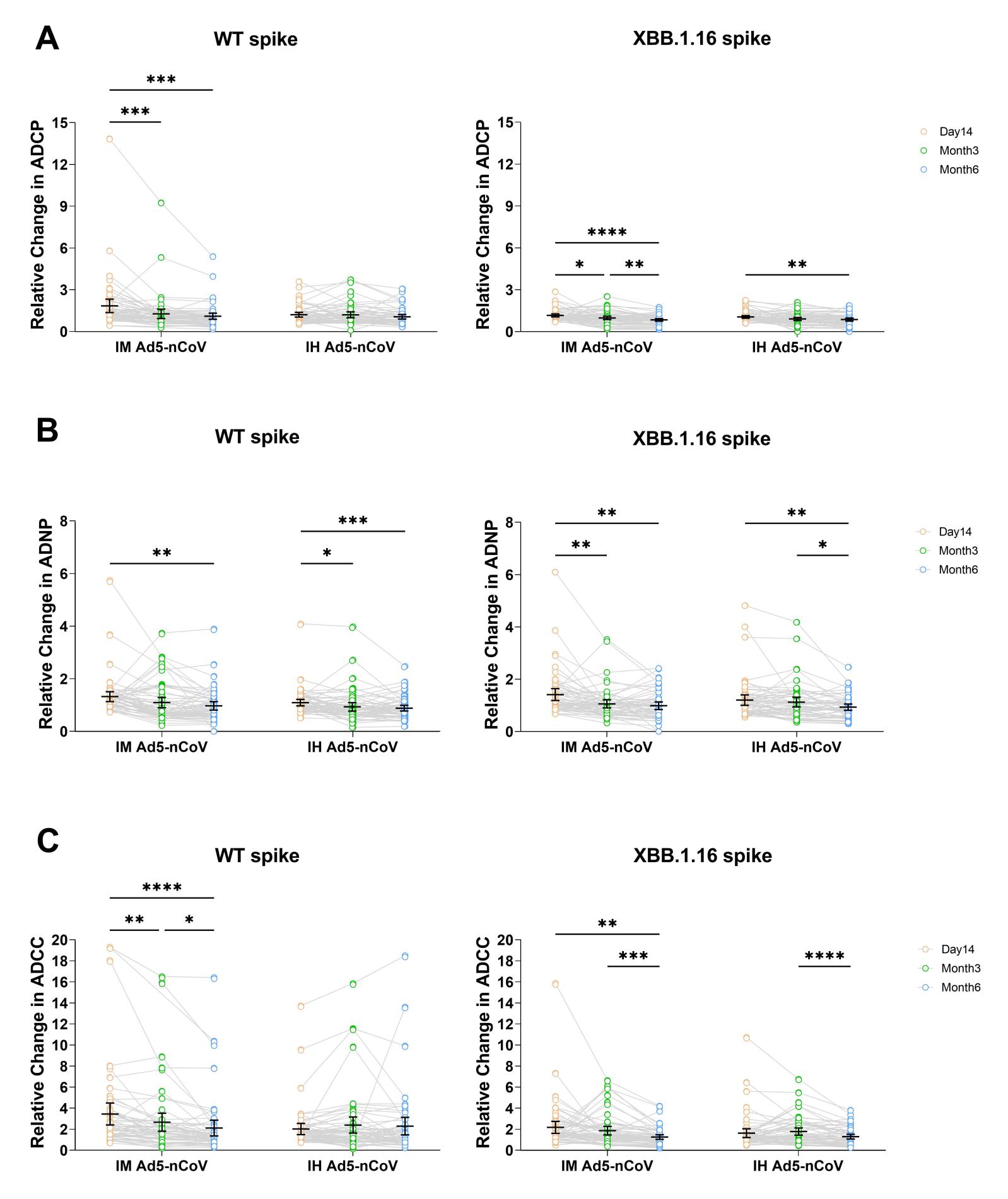


Figure S2. **Pseudovirus neutralization antibodies against SARS-CoV-2 and variants in serum before and after a booster vaccination.** Pseudovirus neutralizing antibody titers against wild-type SARS-CoV-2 (A), BA.4/5 variant (B), and XBB.1.16 (C) in the IM Ad5-nCoV and IH Ad5-nCoV group. Statistical comparisons between time points were performed using the mixed-effect models. The p values for multiple comparisons have been adjusted. IM Ad5-nCoV=adenovirus type 5 vectored COVID-19 vaccine through intramuscular injection. IH Ad5-nCoV=adenovirus type 5 vectored COVID-19 vaccine through oral inhalation. Asterisks indicate statistical significance: * p < 0.05, ** p < 0.01, *** p < 0.001, **** p < 0.0001.

Figure S3. **RBD-specific** **IgG antibodies against SARS-CoV-2 and variants in serum before and after a booster vaccination.** RBD-specific IgG antibodies against wild-type SARS-CoV-2 (A) and BA.4/5 variant (B). Statistical comparisons between time points were performed using the mixed-effect models. The p values for multiple comparisons have been adjusted. IM Ad5-nCoV=adenovirus type 5 vectored COVID-19 vaccine through intramuscular injection. IH Ad5-nCoV=adenovirus type 5 vectored COVID-19 vaccine through oral inhalation. Asterisks indicate statistical significance: * p < 0.05, ** p < 0.01, *** p < 0.001, **** p < 0.0001.


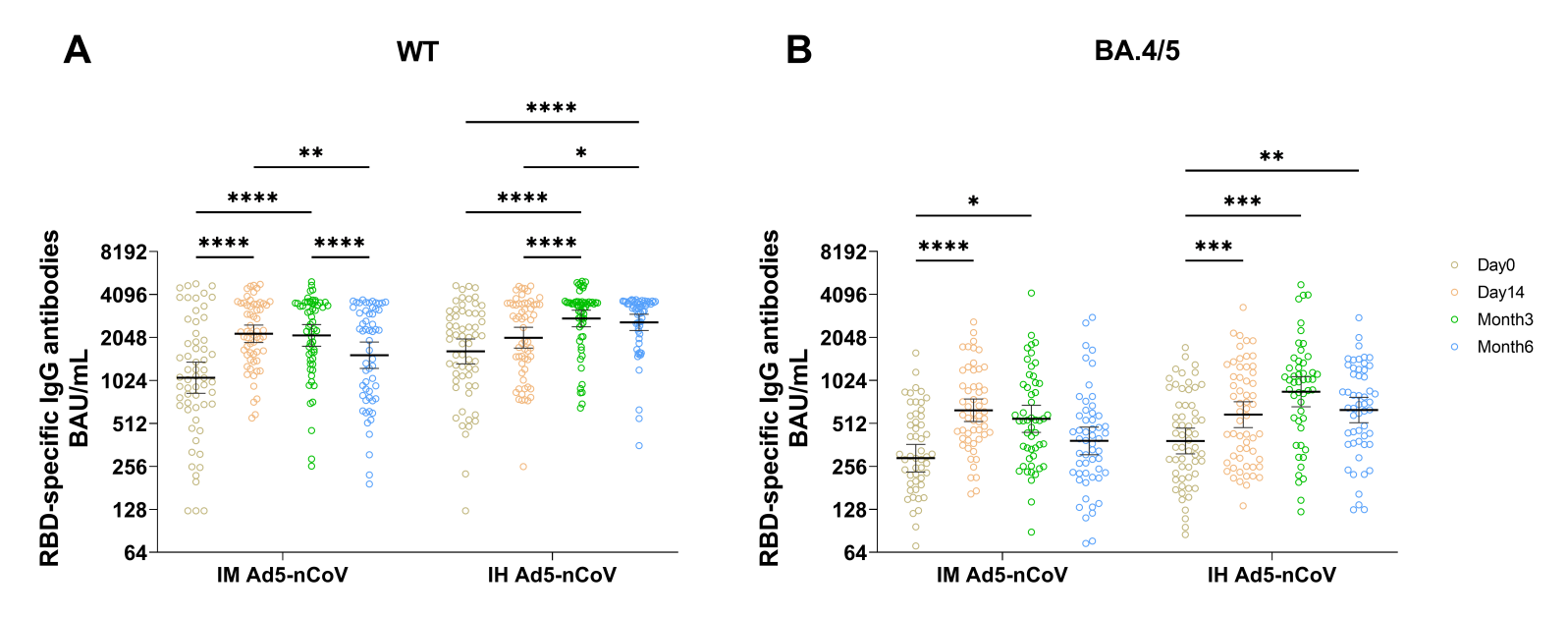


Figure S4. **RBD-specific IgA antibodies against XBB.1.5 variants in serum before and after a booster vaccination.** Statistical comparisons between time points were performed using the mixed-effect models. The p values for multiple comparisons have been adjusted. IM Ad5-nCoV=adenovirus type 5 vectored COVID-19 vaccine through intramuscular injection. IH Ad5-nCoV=adenovirus type 5 vectored COVID-19 vaccine through oral inhalation. Asterisks indicate statistical significance: * p < 0.05, ** p < 0.01, *** p < 0.001, **** p < 0.0001.


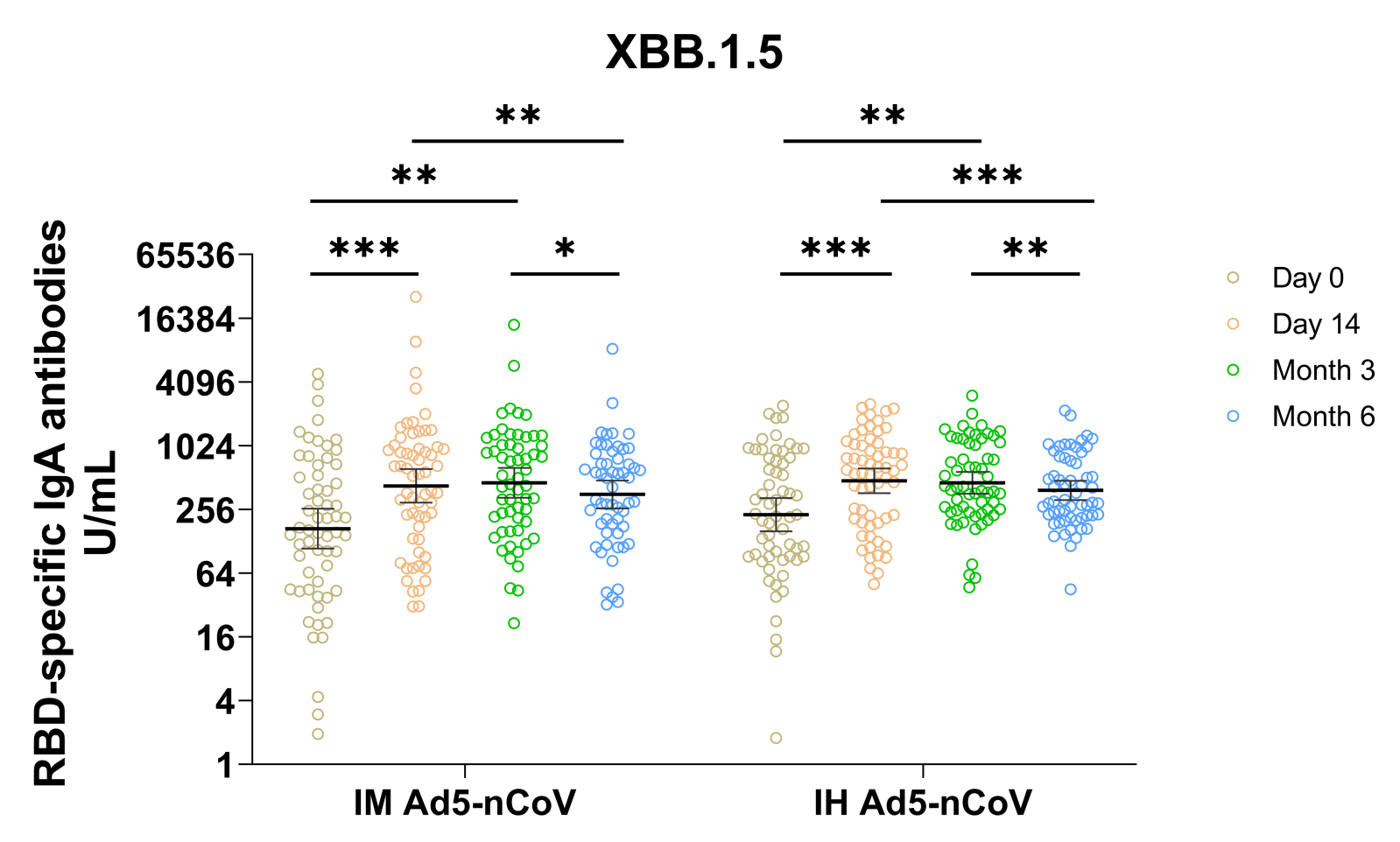

Supplement: Supplementary file 1 [file DataSheet1.docx]
